# Treasures Induced by Narrow-Spectrum: Volatile Phenylpropanoid and Terpene Compounds in Leaves of Lemon Basil (*Ocimum × citriodorum* Vis.), Sweet Basil (*O. basilicum* L.) and Bush Basil (*O. minimum* L.) Under Artificial Light City Farm Conditions

**DOI:** 10.3390/plants14030403

**Published:** 2025-01-29

**Authors:** Anna V. Shirokova, Sofya A. Dzhatdoeva, Alexander O. Ruzhitskiy, Sergey L. Belopukhov, Valeria L. Dmitrieva, Victoria E. Luneva, Lev B. Dmitriev, Victor A. Kharchenko, Azret A. Kochkarov, Elchin G. Sadykhov

**Affiliations:** 1 Federal Research Centre “Fundamentals of Biotechnology” of the Russian Academy of Sciences (Research Centre of Biotechnology RAS), Leninsky Prospect, 33, Build. 2, 119071 Moscow, Russia; sakhapchaeva.1990@gmail.com (S.A.D.);; 2 Federal State Budgetary Scientific Institution “Federal Scientific Vegetable Center” (FSBSI FSVC), Selektsionnaya Str. 14, VNIISSOK Village, 143072 Moscow Region, Russia; 3 Department of Chemistry, Russian State Agrarian University—Moscow Agricultural Academy Named After K. A. Timiryazev (RSAU-MTAA), Timiryazevskaya 49, 127434 Moscow, Russia

**Keywords:** diode light, *Ocimum*, essential oil, phenylpropanoids, isoprenes, capitate glandular trichomes

## Abstract

The cultivation of aromatic plants that are valuable for nutritional and medical aims under artificial conditions with narrow-band LED lighting is becoming widespread. A comparison of the effects of conventional basil field and greenhouse conditions and a city farm (CF) with LED lighting on essential oil and its components was studied in *Ocimum × citriodorum* Vis. “*Kapriz*” (*OcK)*, *O. basilicum* L. “*Queen Sheba*” (*ObQS*) and *O. minimum* L. “*Vasilisk*” (*OmV*). Essential oil (EO) was extracted by hydrodistillation from dry leaves of the basil varieties. EO composition was studied by gas chromatography, while the number of glandular trichomes was studied by scanning electron microscopy. We found that in leaves of CF plants, *ObQS* and *OmV* increased EO yield (22.9 and 22.7 g/kg DW, respectively) compared to field conditions (10.9 and 13.7 g/kg DW, respectively). The number of glands with four-celled heads also increased. In *OcK* plants, the amount of EO was almost unchanged, but the number of capitate glandular trichomes was strongly increased. Biochemical analysis showed that in CF plants compared to field ones, eugenol accumulated 40% more in *ObQS* and three times more in *OmV*. In addition, 10.9% estragol was detected in the leaves of *OcK* plants, which was absent in field plants. Thus, LED lighting conditions increased the biosynthesis of phenylpropanoid volatile components in *Ocimum*.

## 1. Introduction

The components of essential oils are a means of protection that allow plants that synthesize them to resist adverse effects of abiotic factors, grow and maintain the integrity of their population through vegetative and seed propagation. They protect plants from abiotic and biotic factors [1]. Similarly, humans utilize them to produce means of protection against microorganisms and pests, as well as to promote the prolongation of human life (food, medicines, preservatives, protective films, natural pesticides for agriculture) [2,3,4].

Basil (*Lamiaceae* family) is one of the most valuable sources of essential oil, which rapidly forms in significant quantities in above-ground parts, “convenient“ for extraction, in particular in leaves and inflorescences. Many varieties and hybrids of *O. basilicum*, as well as *O. gratissimum* L., *O. canum* Sims., *O. sanctum* L., *O. × citriodorum* Vis., *O. minimum* L., *O. kilimandscharicum* L. and *O. menthaefolium* Hochst. ex Benth., are widely cultivated in the world [5,6,7]. The widespread popularity of basil might originally be due to the fact that its leaves synthesize the same components as well-known spices, cloves (*Syzygium aromacum* (L.) Merr. & L.M.Perry), cinnamon (*Cinnamomum zeylanicum* Blume) and citrus fruits, which during the medieval epidemics of plague and cholera were medicines more valuable than any treasures [8].

*Ocimum* species have diverse biological pharmacological properties such as antibacterial, antiviral, antifungal, antimalarial, antihelminthic, anti-inflammatory, cardioprotective, anti-diabetic, antitumor, chemopreventive, anti-arthritic, anti-dysenteric, sedative and analgesic [9,10,11,12,13,14,15,16,17]. A number of *Ocimum* species possessing anticonvulsant properties are being utilized to treat central nervous system (CNS) disorders, including epilepsy, since approximately one in three patients experience seizures resistant to treatment with currently available anticonvulsants. These sedative and anticonvulsant effects have been experimentally proven for *Ocimum tenuiflorum* L. [18].

For culinary purposes, *O. basilicum* leaves are used fresh and dried to flavor salads, sauces, soups, stews and curries, as well as tea, while seeds are used to cook fresh traditional meals in some regions of Asia such as Iran and India, where basil seeds are frequently included in beverages (Sharbat) and ice desserts (Faloodeh) [19].

The flavor of its leaves and the therapeutic effects of *Ocimum* are due to the essential oil components contained in it. The predominant chemical compounds in essential oil (EO) found in high concentration are a source for physiological forms or chemotypes. Eugenol and methyl chavicol (estragol) chemotypes can be considered the most common in *Ocimum* species [20,21,22,23]. Less common are forms with predominant citral, bergamotene and borneol [24]. A strong lemon odor is characteristic of *O. × citriodorum*, an interspecific hybrid of *O. basilicum* and *O. canum*. The essential oil of this hybrid is rich in a mixture of *trans-citral* (geranial) by up to 31–46% and *cis*-citral (neral) by up to 21–35%, respectively [25].

Substances of essential oils belong to two large groups: terpenes and phenylpropanoids. Volatile phenylpropanoids biosynthesize from shikimic acid (to form the related compounds eugenol, methyl eugenol, methyl chavicol) via the phenylpropanoid pathway. Isoprenes, or terpenoids, form via the mevalonic acid (MVA) pathway, or the 2-*C*-methyl-*D*-erythritol-*4*-phosphate (MEP) pathway, by condensation of two isoprene precursors: isopentenyl pyrophosphate (IPP) and dimethylallyl pyrophosphate (DMAPP) [26,27].

The specific aromatic compounds of basil accumulate in specialized structures—glandular trichomes. Trichomes of plants are sprouts that developed from the epidermis cells of air organs.

According to classification, trichomes fall into two basic types: non-glandular (different hairs, pubescence, etc.) and glandular with apical secretory cells. Trichomes protect plants from abiotic stresses such as water loss, overheating, excessive light and UV radiation, reduce insect damage to plant organs, and provide chemical defense. The structure of trichomes is species-specific, and this trait is usually linked to their function. There are several types of glandular trichomes (GTs), which can be distinguished based on the number and size of cells of their parts [28,29].

In general, glandular trichomes consist of a base, stalk and head of secretory cells. For the classification of GTs, the length of the stalk cells is as important as the size and number of cells and series of the head. There are pilate GTs with a long stalk of one or two very elongated cells and a barrel or spherical unicellular head, and there are peltate GTs with a discoid secretory top attached to the middle of a short stalk, similar to the ancient Greek shield (pelta) in the form of an ivy leaf. Trichomes looking like hairs with a ball or barrel on top and containing a sticky exudate densely cover plants of the family *Solanaceae*. Capitate trichomes have a very short 1–3 short-celled and unicellular stalk and a 2–8-celled spherical or elliptical head. Trichomes without a stalk are called sessile and “capitate glands“ [30]. It is believed that the major contribution to essential oil biosynthesis in basil is made by the large, erroneously called “peltate“ glands, with a 4-celled head. The final number of trichomes on an *Ocimum* leaf formed at an early stage of development does not change during leaf growth, and the density of trichomes decreases with leaf age [31].

*Use of Artificial Light for Plant Cultivation*

It is known that growing conditions such as light intensity, spectral composition, photoperiod and moisture influence the secondary metabolism of plants and, consequently, the yield and composition of essential oils throughout the year. For example, chemical variations in essential oils were related to the season of the year in *O. basilicum* [32]. LED lighting creates new possibilities for growing crops under controlled conditions since LEDs provide monochromatic light that can be combined to create conditions targeted at certain plant species and product purposes [33,34]. Growing demand for natural components of essential oils makes it necessary to study the effects of intensive conditions on economically important species of essential oil crops utilized in industry [35].

In modern “white light“ LEDs for plants, different wavelengths are combined [36,37]. Among other things, the possibility of using a narrow band spectrum allows for the investigation of the effect of single monochromatic light [38]. Cultivation in a controlled environment, where LEDs are generally seen as the most efficient light sources, enhances the concentration of specialized metabolites in herbs, as well as helping to reduce the costs of extraction of active components [39,40].

We aimed to compare the effects of LED and natural lighting on the essential oil component composition, its accumulation and features of glandular trichomes of leaves of three *Ocimum* species.

## 2. Results

### 2.1. Development of Plans in a Field, Greenhouse and City Farm

**Figure 1:**
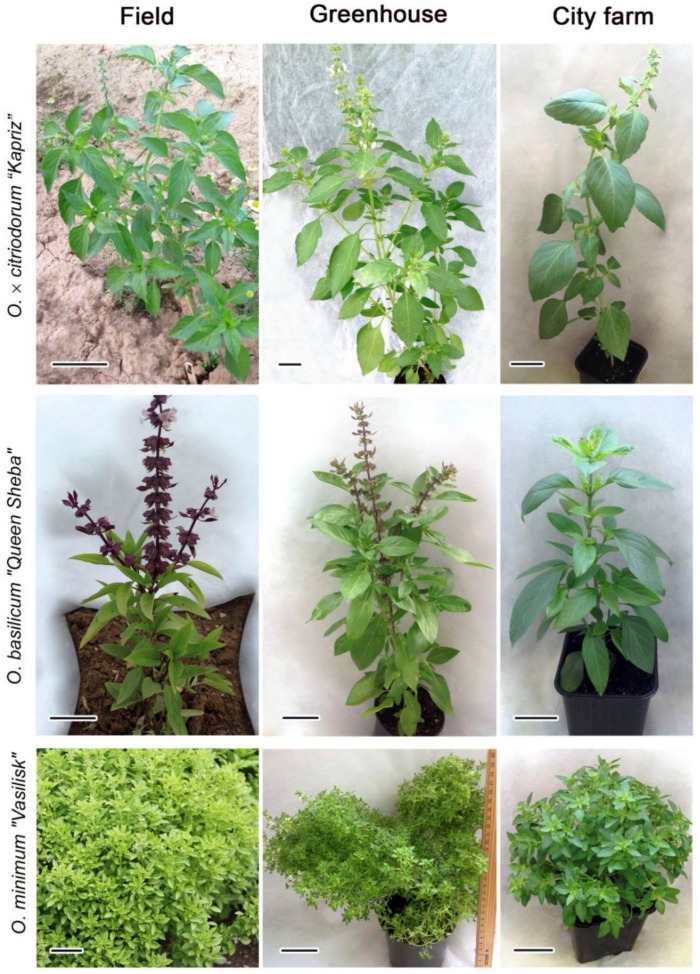
Plants of *Ocimum × citriodorum* cv. “*Kapriz*”, *O. minimum* cv. “*Vasilisk*” and *O. basilicum* cv. “*Queen Sheba*”, grown in a field (F), greenhouse (GH) and city farm (CF).

Plants of all samples exhibited slower growth, more dense branching, and shorter leaves and inflorescences under field (F) conditions compared to greenhouse (GH) and city farm (CF) conditions. In all three locations, plant height and duration of the period before flowering differed dramatically only in *Ocimum × citriodorum* “*Kapriz*” (*OcK*) plants (Figure 1). Field plants were low-growing, compact, with short internodes, their average height being 32.8 ± 3.6 cm, whereas in greenhouse and farm plants it was 61.2 ± 6.5 cm and 36.7 ± 5.7 cm, respectively. In *O. basilicum* cv. “*Queen Sheba*” (*ObQS*), field plants of *ObQS* and CF plants were low-growing—20.4 ± 1.8 cm and 22.3 ± 1.7 cm, respectively, while greenhouse plants were much higher, about 32.6 ± 3.8 cm. In addition, differences in color intensity were observed: shoots, leaf veins and bractlets of field plants were a violet-purple shade, in greenhouse plants they were light mauve, and in farm plants they were even lighter. Plants of *O. minimum* cv. “*Vasilisk*” (*OmV*) had a bigger diameter of above-ground parts in the field than in the greenhouse. The height of field plants was 35.7 ± 4.8 cm, and that of GH and CF plants was 23.6 ± 2.4 and 26.4 ± 2.8 cm, respectively.

### 2.2. Yield of Essential Oil

**Figure 2:**
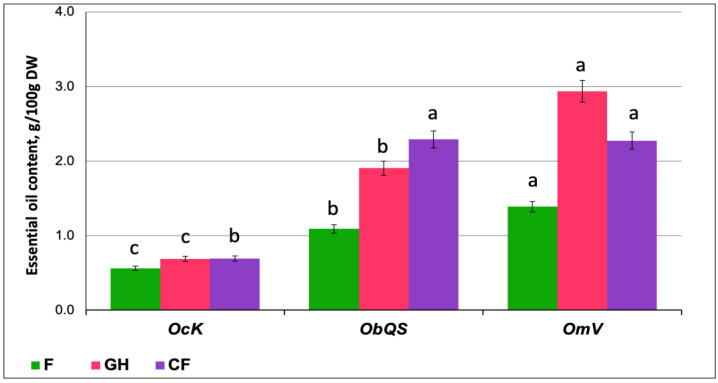
Essential oil content in basil leaves of *Ocimum × citriodorum* cv. “*Kapriz*”, *O. basilicum* cv. “*Queen Sheba*” and *O. minimum* cv. “*Vasilisk*” in the field (F), greenhouse (GH) and city farm (CF). Different letters denote the significant variations measured by Duncan’s multiple range test at *p* < 0.05.

In all studied species, the content of essential oil in the leaves of greenhouse plants was higher than in the field ones (Figure 2). In *OcK* leaves in all locations, the EO content was lower than in other species (up to 0.8 g/100 g DW). *OmV* leaves were found to be richest in EOs, especially when grown in a greenhouse—by about 3 g/100 g DW. The EO content in *OmV* and *ObQS* farm plants was the same, exceeding 2 g/100 g DW. The differences between the minimum and maximum amounts of EO in the *OmV* and *ObQS* samples were approximately twofold.

### 2.3. Component Compositions of EOs

In the studied *Ocimum* species, the component composition of EO was different (Table 1). The lemon scent of the *OcK* leaves was due to a mixture of *trans*-citral and *cis*-citral. In *ObQS* and *OmV* plants, the main compounds were linalool and eugenol.

In CF plants of *OcK*, compared with field plants, the total amounts of the EO major terpene components, that is geranial and neral, remained at the same levels—60% and 58%, respectively. But there was a change in their ratio in the leaves of CF plants, where geranial content decreased and neral increased; as a result, the difference in their amounts was almost 14% (2/5). And on the other hand, only the content of neral increased in GH plants, but the geranial content remained the same as that of field plants. The changes in the content of other EO components were more significant. In CF plants of *OcK β*-caryophyllene, *α*-bergamotene and *α*-bisabolene contents decreased twice compared to those in the field plants, and a quarter decrease occurred in the content of *α*-caryophyllene and germacrene *D* and germacrene *B*.

In *ObQS* plants from the CF compared with those of the field, the main monoterpene component linalool decreased slightly (2.7%). However, the amount of linalyl acetate dropped by 13 times, *β*-myrcene by two times, and germacrene B decreased by a quarter. Geranial, neral, *γ*-cadinene and *γ*-muurolene did not accumulate. On the contrary, germacrene D and muurolol began to accumulate (2% or more), and the amount of α-bisabolene increased fourfold in CF plants. The amount of *α*-bergamotene increased slightly (about 1/5).

In *OmV* CF plants, compared with field ones, the content of most terpenes decreased markedly. Thus, the concentration of the main terpene, linalool, reduced by 11%. The amount of germacrene D reduced by 4.1%, the concentration of α-bulnesene reduced significantly by 1.3%, down to 0.9%, and that of muurolol decreased from 3% to 1.6%. The contents of β-ocimene, 1,8-cineole and α-bergamotene reduced by approximately 1–2%.

Under greenhouse conditions, the contents of some individual compounds in the leaves were intermediate between those found in the field and CF plants like *OcK*, or the highest compared to the other two locations, such as terpinene-4-ol in *OmV* plants. Compounds that were absent under other conditions were also found, for example, bicyclogermacrene 4.1% (*OcK*), 2.1% geraniol (*ObQS*) and *γ*-muurolene (*ObQS*, *OmV*).

The amount of phenylpropene EO components increased significantly in the leaves of CF plants of all species, compared with those in field ones. Thus, eugenol content increased by ¾ in *ObQS* leaves, and in *OmV* plants, it more than doubled. At the same time, the content of methyl eugenol in *ObQS* increased by 14.5 times. In *OcK* plants, estragole (10.9%) was detected, which was not present in the leaves of field plants, and in GH ones, its content was 2.8%.

### 2.4. Features of Glandular and Non-Glandular Trichomes on Leaf Surfaces of OcK, ObQS and OmV

**Figure 3:**
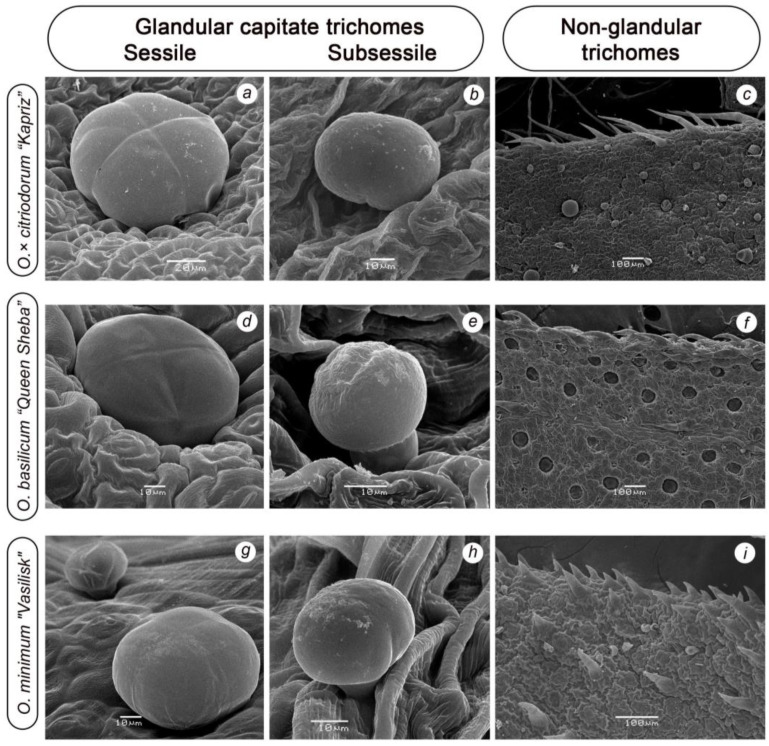
Glandular and non-glandular trichomes on leaves of *O. × citriodorum* cv. “*Kapriz*” (**a**–**c**), *O. basilicum* cv. “*Queen Sheba*” (**d**–**f**) and *O. minimum* cv. “*Vasilisk*” (**g**–**i**). Non-glandular trichomes along leaf edge: (**c**)—multicellular falcate conical hairs; (**f**)—rough trichomes; (**i**)—unicellular short rough trichomes.

Using scanning microscopy, glandular capitate trichomes of two types were discovered in the studied *Ocimum* species (Figure 3): large sunken sessile glands (SGTs) with a large 4-celled secretory head without a stalk (Figure 3a,d,g) and little subsessile (SbGT) ones with a small 2-celled spheroid (Figure 3e) or ellipsoid (Figure 3b,h) head and unicellularity, but a noticeable stalk from an elongated cell. In the projection from above, large glands looked like flat buttons, sunken into the epidermis of the leaf, that is, top cells were nearly at the same level as the epidermis (Figure 3c,d), but the photo of a side projection revealed them to be hemispherical (Figure 3a,g). It was found that SGTs were located throughout the leaf surface on both sides, and SbGTs were located on the surface and on the veins. Non-glandular trichomes served as a taxonomic trait, being species-specific and thus aiding in their identification. All samples showed non-glandular trichomes (NGTs) on the edges of their leaf blades (Figure 3c,f,i). Also, non-glandular trichomes were located on the veins or surfaces of leaves. In *OcK*, soft, long, straight and falcate hairs with a length from 150 μm to 2.5 mm are clearly visible along the veins on both sides. In *ObQS*, short trichomes (to 100 μm) were sparsely located on the veins of the abaxial sides, and the leaves of *OmV* were also covered with short hairs on the adaxial side and on the veins of the abaxial sides. On the leaves of CF plants, the leaf edge trichomes were shorter and sparser than on the leaves of field plants. F, GH and CF plants were found to differ in diameter, shape, location and ratio of GT types on the adaxial and abaxial surfaces of the leaf.

Differences in SGT diameters were particularly noticeable between field plants of all species. Between CF plants, differences in *ObQS* and *OmV* were negligible (Table 2). The largest SGTs (81–95 μm) in field plants were found in *OcK*, and the smallest (53–64 μm), in *OmV*. Large-leaved *Ocimum* species had SGTs with smaller diameters in CF plants compared to F and GH plants, while small-leaved species displayed the opposite trend, having larger diameters of SGT in CF plants. The most noticeable differences in SGT diameter between open-field and CF plants were noted in *OcK* (up to 17% on average), while in *ObQS*, they were about 15%. When comparing the species *O. basilicum* and *O. minimum* with a similar EO component composition, it turned out that the differences in the diameters of sessile glands were more significant (about 15%) between plants under outdoor conditions, while in CF plants, these differences were absent. In greenhouse plants of all cultivars, the variation by diameter in SGT was greater than under CF and field conditions. SbGTs turned out to be 2.5–3 times smaller in diameter than SGTs. Their average diameters for all three samples under all conditions ranged from 25 to 32 μm.

**Figure 4:**
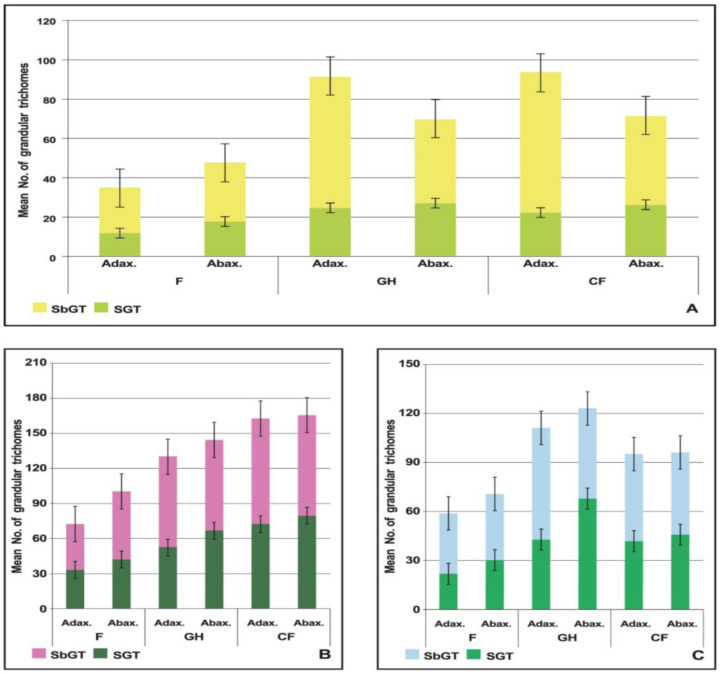
Mean numbers of capitate sessile and subsessile GTs on adaxial (Adax.) and abaxial (Abax.) sides of leaves of *O. × citriodorum* “*Kapriz*” (**A**), *O. basilicum* “*Queen Sheba*” (**B**) and *O. minimum* “*Vasilisk*“ (**C**).

Overall, *O. × citriodorum* had the lowest SGT density per unit area on both leaf sides, whereas *O. basilicum*, the highest among the accessions studied (Figure 4). In *OcK* field plants, there were approximately equal numbers of SGT and SbGT on the adaxial side. In contrast, CF plants had noticeably more SbGT.

In addition, the number of SbGTs, which in field plants sat on the veins, sharply increased, while in CF plants, the constellation of SbGTs were scattered over the entire surface of the adaxial side. As a result, their ratio was 1:2–1:6 in different parts of the leaves. In CF *O. basilicum* plants, the gap between the number of SGTs on the abaxial and adaxial sides was reduced. In *OmV* plants, the largest number of SGTs was observed on the leaves of GH plants, in the peripheral areas and at the base, on the abaxial surface, and SbGT was larger on the adaxial side and along the edge, among NGTs. The scanned images demonstrate that the surfaces of CF plant leaves were characterized by a greater quantity of EO trichomes. In contrast, field plants displayed a high proportion of empty and damaged trichomes.

## 3. Discussion

*Ocimum* species are well known for their special medicinal and aromatic properties. The peculiar aroma of *Ocimum* plants is due to a specific combination of EO volatile compounds, mainly belonging to the classes of terpenoids and/or phenylpropanoids accumulated in specialized epidermal secretory structures called glandular trichomes. They are made up of secretory cell(s) containing the enzymatic machinery for volatile compound biosynthesis and an oil sac for storage [41].

We use the term “sessile” and avoid the term “peltate” to refer to these GTs because peltate GTs have a prominent stalk and disc-shaped head [42], while in the basil samples we studied, such traits were absent (Figure 3a,d,g); on living leaves, sessile GTs look like large drops sunken in the epidermis of the leaf. In *O. × citriodorum*, which has a low EO yield and is dominated by citral (about 60%), SGTs are larger in cross section, but are significantly fewer in number on leaves than in eugenolic species. There may be a relationship between the high citral content and low yield of EOs (usually less than 1%), and in various plant species belonging to *Lamiaceae* fam. and others such as *Pelargonium crispum*, *Lippia citriodora* Kunth., *Melissa officinalis* L., *Dracocephalum moldavica* L. [43,44,45,46,47,48]. In Lemon Basil, under CF conditions, the SGT density increased, with EO content growing by 40%, less significantly than in the other two species.

In our experiment, it was from the leaves of the GH and CF plants that a higher yield of EO was obtained, due to higher numbers of sessile 4-celled glands in them compared to the field plants. Also, sessile glands of this type were abundant on both sides of the leaves in the CF *ObQS* and *OmV* plants, while in F plants, 4-celled sessile glands were mostly concentrated on the lower surface. (Figure 4B). *Mentha piperita* L. [49] and *Ocimum basilicum* were found to have new GTs constantly forming at the early stage of leaf development, which resulted in glands of different ages coexisting in the growth zones [50]. Especially many SGTs of different diameters and ages were observed under CF conditions in *ObQS* leaves.

Capitate GTs of a different type are likely to contribute to the total amount of volatile compounds. Numerous small SbGT *O. × citriodorum* with a 2-celled head, whose diameter was 2.5–3 times smaller than that of sessile GTs, formed in particular abundance in CF plants *OcK*, with ratios of SGTs:SbGTs of 1:4–1:6 observed mainly on the marginal parts of the adaxial sides of leaves, not along the veins or on their surfaces. Histological analysis of capitate GTs with a short stalk and unicellular or bicellular head, described for *Lippia citriodora* and the genus of *Phlomis* (fam. *Verbenaceae*) [51] and several species of *Lamiaceae* fam., demonstrated that they also contained terpenes, as well as lipids, flavonoids and other compounds [52], providing plants with defense against insects and pathogens.

*Biochemical Changes in Basil Plants*

The main effect of LED lighting was on the accumulation of phenylpropene components of EO—methyl chavicol (estragole), eugenol and methyl eugenol (Table 1). Thus, methyl chavicol, otherwise either entirely absent, as was the case in field plants, or below 3% in GH plants, was found in *O. × citriodorum* leaves in quantities as high as 10% under the CF condition.

It has been shown that the primary sites of methyl chavicol biosynthesis are young developing tissues of leaves of the lateral shoots of adult flowering plants. Accumulation decreases as leaves develop [53].

Data on the influence of external factors on the accumulation of methyl chavicol and methyl eugenol in basil leaves are contradictory. For instance, it was demonstrated that in *O. basilicum* at the flowering stage, the accumulation of methyl chavicol and methyl eugenol, along with *β*-myrcene and *α*-bergamotene, enhanced stress caused by drought [54].

Another study evaluated the effect of water deficiency on the accumulation rates of methyl chavicol and methyl eugenol and the expression profiles of five critical genes of their biosynthesis (*4Cl*, *C3H*, *COMT*, *CVOMT* and *EOMT*) in three Iranian cultivars of *O. basilicum*. Increases in the contents of these components were detected in only two of the three varieties, and all the studied genes showed different transcription coefficients, which indicates the dependence of such a reaction on internal varietal characteristics [55]. On the other hand, in the leaves of methyl chavicol and eugenol varieties in the greenhouse, under conditions of decreased illumination, lower temperature and increased humidity (autumn cycle), methyl eugenol accumulated in large quantities [32].

At the same time, in our experiment, methyl eugenol accumulated in noticeable amounts only in the leaves of *O. basilicum* cv. “*Queen Sheba*” under sufficient illumination CF (2.9%), providing further evidence of the influence of varietal characteristics. Lewinsohn E. et al. (2000) showed that the linalool-eugenol chemotype SW of *O. basilicum*, completely devoid of methyl chavicol or methyl eugenol, lacked the activity of chavicol-*O*-methyltransferase (CVOMT) and eugenol-*O*-methyltransferase (EOMT), which explains the absence of p-methoxylated allylphenols in this chemotype [56]. However, in our case, the unexpected “manifestation” of methyl chavicol indicated that the activity of chavicol-*O*-methyltransferase (CVOMT) may occur in vitro. Additionally, the return to estragole accumulation may confirm the hybrid nature of *O. × citriodorum*, since the estragole chemotype is common among *Ocimum basilicum* cultivars [57]. Interestingly, from an investigation into the genetic diversity in the whole genus *Ocimum* and genetic relationships among hybrid accessions by the method of cluster analysis, within a single clade of the samples k2.1 115 (“*Sweet Dani*”), with citral (68%) and 116 (PI 172996), essential oil consisting of 91% methyl chavicol was grouped [58].

Changes in the relative content of essential oil components from different classes of volatile compounds are attributed to increased accumulation of phenylpropenes in all chemotypes, including terpene (citral) *OcK* and “mixed” eugenol-linalool.

This may be due to activity of enzymes in the phenylpropanoid pathway. The immediate precursors of the shikimate pathway are phosphoenolpyruvate (PEP) and d-erythrose-4-phosphate (E4P), which come from glycolysis and the pentose phosphate pathway (PPP), respectively. They also provide precursors for the MEP and MVA pathways, and thus, the latter compete for carbon allocation between the “terpene pathways” and the shikimate/phenylpropanoid pathway [59]. The rate of biosynthesis of any particular volatile compound is not only controlled by the amount of substrate available, but is also determined by the activity of the enzymes responsible for the final stage of its formation [60].

Gurav T.P. and colleagues (2022) showed that in the citral-rich SD line of *O. basilicum*, high activity of MEP pathway enzymes and decreased carbon flux into the phenylpropanoid pathway, as well as higher levels of terminal enzymes of the terpenoid biosynthetic pathways along with low levels of PAL, resulted in a terpenoid-rich chemical profile. In another EMX-1 line, the increased content of methyl chavicol (estragole) was due to a high level of PAL activity [57]. Thus, directing carbon flux through the overexpression or repression of critical enzymes from entry points, key intermediates or endpoints of either the phenylpropanoid or terpenoid pathway may modify key chemotypes in *Ocimum* species.

**Figure 5:**
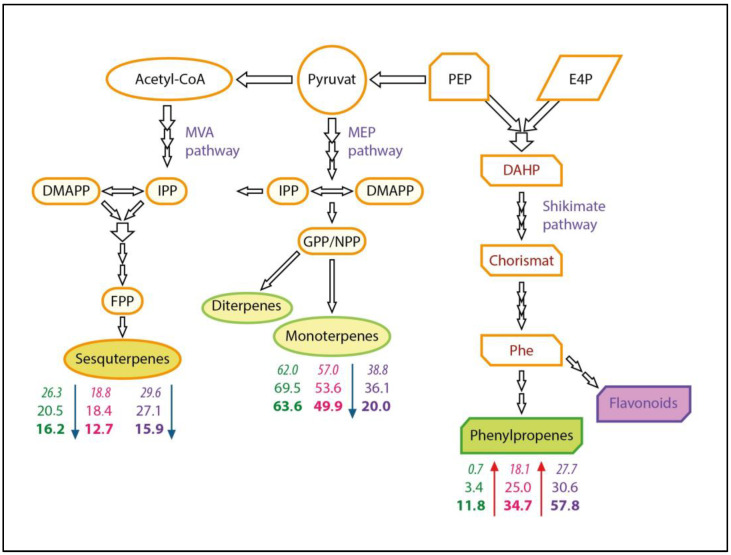
Changes in volatile compounds in the leaves of *O. × citriodorum* “*Kapriz*”, *O. basilicum* “*Queen Sheba*” and *O. minimum* “*Vasilisk*” under outdoor and indoor conditions. Designation: *OcK*, *ObQS*, *OmV*; in top-down columns—*field*—*italic*, greenhouse—normal, city farm—**bold**. The arrows next to the numbers show an increase (red) or decrease (blue) in component content from different classes under city farm conditions compared to the field [59], with changes.

In our experiment, we apparently observed a switch in carbon flux towards the phenylpropanoid pathway and an increase in the level of its products, estragole, eugenol and methyleugenol, as well as a decrease in the content of terpenes of the MEP and MVA pathway (Figure 5).

At the same time, a decrease in the activity of flavonoid biosynthesis enzymes, which led to a markedly reduced formation of anthocyanins, caused an increase in the accumulation of phenylpropene volatile compounds, with a slightly lower content of terpenes. For example, in field plants *Ocimum basilicum* “*Queen Sheba*”, with deep purple stems, leaf petioles and bracts, part of the precursors served as a material for the biosynthesis of anthocyanins, while under CF conditions, their formation plummeted (Figure 1). As a result, the increase in eugenol content occurred without a noticeable decrease in linalool and other mono- and sesquiterpenes. A similar dependence was revealed under the spectrum with a minimum share of blue color and a maximum share of red color, which increased the content of phenolic acids and anthocyanins and caused the content of volatile compounds to decline [61].

When comparing the effects of different light ratios of blue:red:white, with the complete absence of blue (0:5:5) and with its presence (2:3:5), blue light was shown to increase the yield of essential oil in Sweet Basil, in which methyl cinnamate, a phenylpropene component, predominates [62].

Summing up, comparison of the effect of exposure of basil plants to various combinations of LEDs showed that blue light in combination with red enhances the accumulation of secondary metabolites, and in some cases organic acids and anthocyanins, while in others, volatile compounds [37,63].

On the other hand, in *Melissa officinalis* L., exposure to different LEDs did not cause a significant increase in EOs composed of terpene compounds, as in *O. × citriodorum*. The content of essential oil in two genotypes of *M. officinalis* under illumination with a mixture of red (660 nm) + blue (460 nm) in a ratio of 70:30 remained at the same level as in plants in the greenhouse (in the range of 0.27–0.32%). However, the concentrations of the main monoterpenes, citral, citronellal and linalool, increased by a quarter to a half under red-blue light conditions. At the same time, the amount of sesquiterpenes, depending on the compound, either increased, went down or remained at the level of greenhouse plants [64]. That is, changes in the citral chemotype of *O. × citriodorum* were similar to changes in the terpene content in *M. officinalis*. Also, under LED conditions, it was possible to stimulate the accumulation of both phenylpropene volatile components, which easily respond to changes in conditions, and individual terpenes.

## 4. Materials and Methods

### 4.1. Plant Material and Growing Conditions

Seeds of cultivars *Ocimum basilicum* “*Queen Sheba*“, *O. × citriodorum* “*Kapriz*“ (developed by FSBSI FSVC) and *O. minimum* “*Vasilisk*“ (developed by “Gavrish“ company) from the collection of the Research Centre of Biotechnology RAS were used. Work was carried out in a vertical city farm with artificial light provided by the Research Centre of Biotechnology RAS, while field and greenhouse plants were grown in west of the Moscow region in FSBSI FSVC.

In *O. × citriodorum* “*Kapriz*“, the leaves were light green, with a salty tinge and a strong smell of lemon. In *O. basilicum* L. “*Queen Sheba*”, the leaves were narrow, green and purple-streaked with a strong smell of clove. In *O. minimum* L. cv. “*Vasilisk*“, bushes were thick and leaves were small and green, with a light smell of clove.

Plant seeds were sown in peat substrate in early April. The plants included in the control group were grown in a cassette (64 cells). A total of 35 young plants of each plant variety at the age of 50 days were planted on an experimental plot (“field”) in two lines (double row planting), 25 cm between plants and 30 cm between lines, and grown according to the common method.

For the greenhouse and vertical artificial light city farm, 3-day seedlings were pitched into pots of 9 cm. A total of 30 plants of each plant variety were placed in the glazed greenhouse without lighting and 30 plants of each sample were placed in the vertical city farm with artificial LED lighting.

The traits of plant growth and development were evaluated in the phases of six pairs of leaves on the main shoot (flower bud formation). The heights of plants from their cotyledons to their main inflorescence, lengths and widths of the fourth and fifth pairs of the leaf lamina, and numbers of lateral shoots were taken into account.

Light was provided by Plant Lighting (Edison Opto Corporation Ltd., New Taipei City, Taiwan). The characteristics of the LEDs were 450 nm blue (B), 660 nm red (R), 700–800 nm deep pink (DP) and 4000 K white (W). The flux density of photosynthetic photons irradiating a surface (PPFD) were set at 270–280 μmol/m^2^/s. Fluence rates were thus B:R:W:DP, as 25:25:80:20. The photoperiod was 14/10 h (light/dark). The temperature in the CF was 24–26 °C.

### 4.2. Essential Oil Isolation and Component Identification by GC

In the flowering phase, side shoots were cut off and dried to leaf breakage at 23–25 °C on laboratory tables. For hydrodistillation, only leaves were used. Material was placed in a 500 mL round-bottom flask, distilled water was added (1:2) and the mixture was distilled for 60 min. Essential oil was isolated using Ginsberg’s collector. The obtained essential oil yield was collected into vials, weighed and calculated in 1 kg^−1^ of dry plant material. Essential oils were isolated in the Department of Chemistry of the Timiryazev Agricultural Academy.

### 4.3. Determination of the Compositions of Volatile Compounds of Basil Samples

The compositions of the essential oil components of the three samples were studied by gas chromatography in extracts from fresh leaves from five plants (n = 5). Weighed portions of leaves (10 g) from shoots of 1st and 2nd orders of branching were placed in glass jars (large leaves were cut into sections) and immersed with *MeOH*/hexane/benzene in equal volumes (extraction mixture 1:2 *w*/*v*). The extraction was carried out at a temperature of +4 °C, for no less than 48 h. Then, the filtered extract was added to an equal volume of distilled water, and after the complete separation of phases, the upper phase was selected in vials for research on a Shimadzu GC 2010 Plus chromatograph with a GCMS-QP2010 Ultra quadripartite sample (Japan). Chromatographic separations were performed on 30 m × 0.25 mm, d_r_ 0.25 µm MDN-5 capillary column (Supelco). Helium was used as carrier gas at flow rate of 1 mL/min (36,5 sm/s), split 1:10. Injector temperature was 180 °C, interface temperature was 205 °C, detector temperature was 200 °C. The following GC oven temperature parameters were applied: 60 °C for 2 min, 5 °C/min to 120 °C, 10 °C/min to 150 °C, 30 °C/min to 300 °C, 300 °C for 2 min. Mass recording range was from 29 to 400 *m*/*z*. The identification of peaks was performed using the NIST 11 Mass Spectrum Library.The compounds of the volatile extracts were identified by comparing their mass spectra with those of internal reference libraries (NIST 11). The biochemical analysis was carried out in the FRC FB RAS laboratory.

### 4.4. Scanning Electron Microscopy (SEM)

To obtain microphotographs of the adaxial and abaxial sides of young fully expanded leaves and individual trichomes, the leaves were cut from each of the six flowering plants. *O. × citriodorum* “*Kapriz*” and *O. basilicum* “*Queen Sheba*” were studied by their six-leaf laminas (15 and 25 mm long, respectively) from first-order shoots. For *O. minimum* “*Vasilisk*”, 16 leaves were cut from third-order shoots of each plant. The fresh leaves were cut into pieces (about 3–5 mm long by 3–5 mm wide) and immersed in 2% (*v*/*v*) glutaraldehyde (GA) in phosphate-buffered saline (PBS) at pH 7.2 for 5 days, then washed twice in phosphate-buffered saline, with changing of the buffer every wash. After that, the material was immersed into 30, 50, 70 and 96% (*v*/*v*) alcohol solutions, and stored in 96% alcohol for three days. Then, before SEM, the leaf pieces were passed through acetone to ensure the complete removal of water and subjected to critical point drying (Hitachi, Japan). The leaf fragments were then mounted on stubs with double-sided adhesive carbon strips (Double Sided Carbon Tape, 8 mm × 20 mm) and sputter coated with gold (Au coating thickness—20 nm) using IB-3 Ion Coater (Eiko Engineering Co., Hitachinaka, Japan).

The adaxial and abaxial leaf surfaces were examined using a JSM-6380LA scanning electron microscope (JEOL Technics Ltd., Tokyo, Japan) at 20 kV accelerating voltage. Digital images were captured at various magnifications (×100 to ×5000) at a 10 kV accelerating voltage and a stub distance of 10 mm. Trichomes were counted on the adaxial and abaxial sides of parts at the magnifications of ×70–×200 μm. The lengths of the hairs and diameters of glandular trichomes were measured using SEM Control User Interface software Version 7.11 (JEOL Technics Ltd., Tokyo, Japan). The numbers and diameters of glandular trichomes on the surfaces of the leaves were calculated by ADF Image Capture ADF PRO 03 ADF Optics CD, Ltd. (Shenzhen, China, universal stereomicroscope ADF S645). The average number of both types of glandular trichomes was calculated on a 10 mm^2^ area of 80 parts of each plant variety.

### 4.5. Statistical Analysis

The results are presented as the mean of three replicates ± standard error (SE). Differences between treatments for the different measured variables were tested by one-way analysis of variance (ANOVA), followed by Duncan’s test, with significant differences found (*p* < 0.05) using XLSTAT software (version 2014.5.03).

## 5. Conclusions

In CF plants of *O. basilicum* with the linalool-eugenol chemotype, an almost twofold increase in the content of phenylpropanoids was accompanied by a moderate decrease in terpene components, while in *O. minimum* (eugenol-linalool chemotype), a significant decrease in both monoterpene and sesquiterpene compounds was observed, along with a threefold increase in the amount of phenylpropanoid EO components. Surprisingly, in *Ocimum × citriodorum*, which contained almost only terpene compounds, under CF conditions, a noticeable amount of the phenylpropanoid volatile component (estragole) was also formed.

It is probably premature to draw conclusions about the positive or negative impact of the CF condition based on the study of one lighting mode and three varieties, one of which was radically different from the other two in terms of the composition of the main components, and the other two belong to different species. However, one thing is certain—under CF conditions, the compositions of the components changed, the amount of terpene components decreased, the taste became more pungent and the fragrances of eugenol-containing samples lost their diversity as the concentration of eugenol increased in *ObQS* and *OmV* to 32% and 57%, respectively. Eugenol, even in minimal amounts, imparts a strong flavor and aroma. With a significant increase in its concentration, its clove-like scent overshadowed all other components. Thus, the use of narrow-wave radiation generated by LEDs allows for the use of this lighting method to obtain raw materials with a high content of essential oil and a predominance of phenylpropene compounds.

## Figures and Tables

**Table 1:** The compositions of EOs of *Ocimum × citriodorum* cv. “*Kapriz*“, *O. basilicum* cv. “*Queen Sheba*” and *O. minimum* cv. “*Vasilisk*” grown in the field (F), a greenhouse (GH) and a city farm (CF), %.

Compound	*O. × c.* “*Kapriz*”			*O.b.* “*Queen Sheba*”			*O.m.* “*Vasilisk*”		
	F	GH	CF	F	GH	CF	F	GH	CF
	Area, %								
*β*-Myrcene	0.0	0.0	0.0	**2.1**	**1.7**	**0.9**	0.3	0.5	0.2
1,8-Cineole	0.0	0.0	0.0	11.6	11.9	12.0	**7.3**	**8.1**	**5.4**
*β*-Ocimene	0.0	0.0	0.0	**1.3**	**1.9**	**1.0**	**3.7**	**4.5**	**2.4**
Linalool	0.3	0.0	0.7	**28.9**	**26.2**	**27.8**	**18.2**	**12.5**	**7.2**
Terpinene-4-ol	0.0	0.0	0.0	0.0	0.0	0.0	1.0	**2.3**	**0.9**
Estragole	**0.0**	**2.8**	**10.9**	0.0	0.0	0.0	0.0	0.0	0.0
*cis*-Citral (Geranial)	25.7	25.7	23.0	0.2	0.6	0.0	0.0	0.0	0.0
Geraniol	0.0	0.0	0.0	0.0	**2.1**	0.0	0.0	0.0	0.0
Linalyl acetate	0.0	0.4	0.9	3.9	0.0	0.3	0.0	0.0	0.0
*trans*-Citral (Neral)	**32.2**	**38.7**	**36.8**	0.3	0.9	0.0	0.0	0.0	0.0
Eugenol	0.7	0.7	0.7	**17.9**	**24.8**	**31.8**	**27.7**	**30.4**	**57.4**
*β*-Elemene	0.8	0.0	0.6	0.3	0.4	0.4	**1.9**	**1.1**	**0.8**
Methyleugenol	0.0	0.0	0.0	0.2	0.2	**2.9**	0.0	0.2	0.4
*β-*-Caryophyllen	6.0	3.6	3.2	0.5	0.6	0.4	0.2	0.3	0.0
*α*-Bergamotene	**1.4**	**1.1**	**0.7**	3.3	3.8	3.8	5.0	4.4	4.0
*α*-Caryophyllene	1.8	1.3	1.3	0.0	0.3	0.2	0.9	0.8	0.5
Germacrene *D*	5.8	0.0	4.3	0.0	0.8	2.0	**6.8**	**0.7**	**2.7**
Bicyclogermacrene	0.0	**4.1**	0.0	1.8	1.9	0.0	0.0	0.0	0.0
Germacrene *B*	**2.1**	**1.7**	**1.4**	**1.2**	1.0	**0.8**	**2.7**	**2.5**	**1.4**
*α*-Bulnesene	0.0	0.5	0.0	0.4	0.7	0.3	**2.8**	**1.9**	**0.9**
*γ*-Cadinene	0.0	0.0	0.0	**2.5**	**2.1**	**0.0**	0.0	0.0	0.0
*α*-Bisabolene	**4.8**	**3.5**	**2.4**	**0.3**	**0.3**	**1.2**	0.0	0.0	0.0
Muurolol	0.0	0.0	0.0	0.0	**3.6**	**2.4**	**3.0**	**0.5**	**1.6**
*γ*-Muurolene	0.0	0.0	0.0	**5.5**	0.0	0.0	0.0	**3.2**	0.0
Minor components	7.8	9.7	6.3	14.4	13.4	8.9	13.6	21.5	8.5

Designation: contrast changes in compound concentration in essentials oil are highlighted bold. The compound classes are highlighted by color: cream—monoterpenes, yellow—sesquitherpenes, light green—phenylpropenes.

**Table 2:** Leaf glandular trichome diameters of field, greenhouse and city farm plants of *Ocimum × citriodorum* cv. “*Kapriz*”, *O. basilicum* cv. “*Queen Sheba*” and *O. minimum* cv. “*Vasilisk*”.

Condition	Glandular Trichome Diameter, μm					
	*O. × citriodorum* cv.”*Kapriz*”		*O. basilicum* cv. “*Queen Sheba*”		*O. minimum* cv. “*Vasilisk*”	
	Sessile	Subsessile	Sessile	Subsessile	Sessile	Subsessile
F	88.1 ^a^ ± 6.9	31.4 ^b^ ± 4.8	73.4 ^b^ ± 6.4	30.5 ^a^ ± 3.6	62.2 ^c^ ± 5.9	22.9 ^b^ ± 1.6
GH	76.5 ^b^ ± 9.0	30.7 ^a^ ± 3.8	70.4 ^b^ ± 6.7	29.7 ^b^ ± 3.9	65.7 ^b^ ± 7.5	26.8 ^a^ ± 2.1
CF	73.3 ^b^ ± 6.4	29.5 ^a^ ± 3.9	62.7 ^c^ ± 4.9	28.0 ^b^ ± 1.7	62.0 ^c^ ± 4.5	28.7 ^b^ ± 3.6

Different letters denote the significant variations measured by Duncan’s multiple range test at *p* < 0.05.

## Data Availability

The original contributions presented in this study are included in the article; further inquiries can be directed to the corresponding author.
